# Ezh2 inhibition in Kras-driven lung cancer amplifies inflammation and associated vulnerabilities

**DOI:** 10.1084/jem.20180801

**Published:** 2018-12-03

**Authors:** Michela Serresi, Bjorn Siteur, Danielle Hulsman, Carlos Company, Matthias J. Schmitt, Cor Lieftink, Ben Morris, Matteo Cesaroni, Natalie Proost, Roderick L. Beijersbergen, Maarten van Lohuizen, Gaetano Gargiulo

**Affiliations:** 1 Molecular Oncology, Max Delbrück Center for Molecular Medicine in the Helmholtz Association, Berlin, Germany; 2 Mouse Cancer Clinic, Netherlands Cancer Institute, Amsterdam, Netherlands; 3 Division of Molecular Genetics and Cancer Genomics Centre, Netherlands Cancer Institute, Amsterdam, Netherlands; 4 Oncode Institute, Utrecht, Netherlands; 5 Division of Molecular Carcinogenesis and Netherlands Cancer Institute Robotics and Screening Center, Netherlands Cancer Institute, Amsterdam, Netherlands; 6 Fels Institute, Temple University School of Medicine, Philadelphia, PA

## Abstract

Kras-driven non–small-cell lung cancers (NSCLCs) are a leading cause of death with limited therapeutic options. Many NSCLCs exhibit high levels of Ezh2, the enzymatic subunit of polycomb repressive complex 2 (PRC2). We tested Ezh2 inhibitors as single agents or before chemotherapy in mice with orthotopic Kras-driven NSCLC grafts, which homogeneously express Ezh2. These tumors display sensitivity to EZH2 inhibition by GSK126 but also amplify an inflammatory program involving signaling through NF-κB and genes residing in PRC2-regulated chromatin. During this process, tumor cells overcome GSK126 antiproliferative effects. We identified oncogenes that may mediate progression through an in vivo RNAi screen aimed at targets of PRC2/NF-κB. An in vitro compound screening linked GSK126-driven inflammation and therapeutic vulnerability in human cells to regulation of RNA synthesis and proteostasis. Interestingly, GSK126-treated NSCLCs in vivo also showed an enhanced response to a combination of nimesulide and bortezomib. Thus, Ezh2 inhibition may restrict cell proliferation and promote defined adaptive responses. Targeting these responses potentially improves outcomes in Kras-driven NSCLCs.

## Abstract (precis)

Kras-driven non–small-cell-lung cancers (NSCLCs) are a leading cause of death with limited therapeutic options. Serresi et al. show that inhibiting Ezh2 in orthotopic *KrasG12D*-driven NSCLC unleashes an inflammatory response rewiring tumor progression and amplifying associated vulnerabilities that could be therapeutically exploited.

## Graphical Abstract

**Figure:**
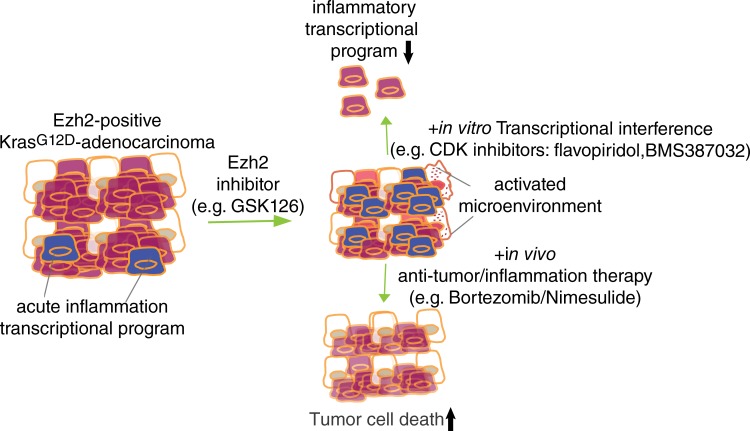

## Introduction

Ras signaling is a major oncogenic driver of human cancers, but there are currently no therapies that effectively target tumors with driver mutations in Ras genes (Vivanco, 2014). Non–small-cell lung cancer (NSCLC) is the most prevalent form of cancer in the western world, and ∼35% of all patients exhibit mutations in Kras, a key component of the Ras pathway (Cancer Genome Atlas Research Network, 2014; Chen et al., 2014).

Ezh2 is the enzymatic component of polycomb repressive complex 2 (PRC2). This complex is responsible for the transcriptional repression of many genes and contributes to the maintenance of cell identities in multiple tissues. To exert these functions, the PRC2 holoenzyme, which also includes nonenzymatic components such as Eed and Suz12, catalyzes trimethylation of lysine 27 on histone H3; this modification in the promoter regions of genes is often a crucial step in their silencing (Margueron and Reinberg, 2011). NSCLCs and many other tumors exhibit Ezh2 overexpression, which is considered oncogenic and is used as a prognostic factor for outcomes in several human cancers. EZH2 has attracted significant interest as a potential target for drugs, because its inhibition would presumably lead to a reactivation of silenced tumor suppressor genes. In NSCLC, it is proposed that when Ezh2 is overexpressed, cells fail to transcribe tumor suppressor genes and microRNAs that would otherwise restrict tumor growth (Friedman et al., 2009).

A global deletion of Ezh2 is embryonically lethal (O’Carroll et al., 2001), but Ezh2 can be depleted in adult animals without causing significant problems: 12 wk of continuous Ezh2 systemic inhibition in adult animals carrying a doxycycline (dox)-inducible shRNA significantly depletes Ezh2 mRNA and protein without causing overt tissue phenotypes (Michalak et al., 2013). The S-adenosylhomocysteine hydrolase inhibitor DZnep proved to efficiently target the enzyme and to impair tumor growth in a subset of NSCLC genotypes with epidermal growth factor receptor (EGFR) or BRG1 mutations when combined with the topoisomerase II inhibitor etoposide (Fillmore et al., 2015). However, DZnep is unlikely to gain momentum as an Ezh2 inhibitor in clinical trials due to significant off-target effects and toxicity (Miranda et al., 2009). Nevertheless, more specific S-adenosylhomocysteine–competitive Ezh2 inhibitors have recently completed preclinical testing successfully (Sneeringer et al., 2010; McCabe et al., 2012).

Multiple synthetic lethal screens conducted to find Kras mutant associated vulnerabilities converged on indicating an important requirement for proteasome activity in Ras mutant solid tumors (Barbie et al., 2009; Luo et al., 2009; Kumar et al., 2012). The proteasome inhibitor bortezomib (BTZ; clinical name Velcade) is approved for use to treat patients with multiple myeloma. BTZ is believed to act through an inhibition of the pro-inflammatory and proto-oncogenic transcription factor NF-κB. Proteasomal degradation of IkBα, an endogenous inhibitor of the pathway that directly interacts with NF-κB to sequester it in the cytoplasm (Demchenko and Kuehl, 2010), is a critical step in the constitutive self-inhibition of the NF-κB found in healthy cells (Arkan and Greten, 2011; Hinz et al., 2012). It has been shown that BTZ treatment of multiple myeloma prevents the degradation of IkBα. Currently, BTZ is being tested in a phase 2 clinical trial in patients with *Kras^G12D^* mutant NSCLC (NCT01833143). However, BTZ alone or in combination with pemetrexed in previous studies did not significantly extend the overall survival in NSCLC patients (Scagliotti et al., 2010), indicating that specific treatment combinations may be required.

NF-κB is a critical promoter of tumor progression, including in NSCLC. In a Kras-driven genetically engineered mouse model reflecting NSCLC biology and response to therapy (Jackson et al., 2001; Johnson et al., 2001; Singh et al., 2010), deletion of NF-κB severely impairs tumor growth (Meylan et al., 2009). Importantly, the proapoptotic response in tumor cells upon pharmacological inhibition of NF-κB appeared to be context dependent in Kras-driven NSCLC models (Meylan et al., 2009; Xue et al., 2011), indicating that additional pathways contribute to modulating NF-κB dependences.

Recently, we showed that PRC2 inhibition by Eed deletion promotes the acquisition of an inflammatory phenotype in a context-dependent manner (Serresi et al., 2016). The link between PRC2 inhibition, inflammation, and Kras-driven tumorigenesis is also found in the pancreas. Loss of EZH2 results in persistent inflammation during pancreatic homeostasis and facilitates *Kras^G12D^*-driven tumorigenesis. Under these experimental conditions, regular dosing with the anti-inflammatory Cox-2 inhibitor nimesulide (NM) preserves tissue homeostasis and delays neoplastic initiation (Mallen-St Clair et al., 2012).

In summary, there is ample evidence supporting the idea that a combination of Ezh2 inhibitors and anti-inflammatory agents might provide an effective treatment for a subset of *Kras^G12D^*-driven NSCLCs. Here, we investigate how orthotopic grafts of *Kras^G12D^*-driven NSCLC cells respond to a clinically relevant Ezh2 inhibitor and investigate the adaptive response mechanisms with a view toward selectively exploiting chemotherapy to target acquired vulnerabilities in a sequential or “neoadjuvant-like” dosing scheme.

## Results

### Ezh2 is important for proliferation of Kras-driven NSCLC cells

**Figure 1.**
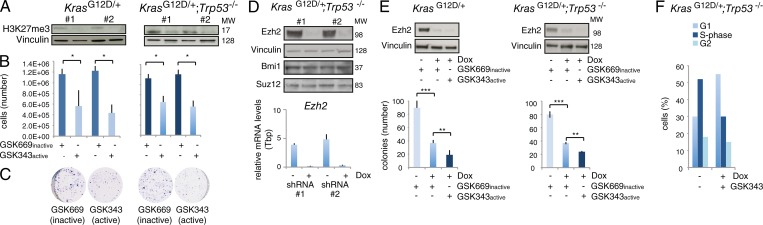
**PRC2 regulates in vitro Kras-driven NSCLC cell proliferation and H3K27me3 in a p53-independent manner. (A)** *Kras^G12D/+^* and *Kras^G12D/+^;Trp53*^−/−^ primary cell lines were generated from mouse NSCLC (two per genotype, #1-#2), and enzymatic EZH2 inhibition was performed with the specific GSK343 compound (100 nM). Immunoblot was performed after 120 h. The inactive GSK669 compound served as a control. **(B)** Cell numbers relative to the cells in A. **(C)** Representative images of colony-formation assays using 500 cells for the indicated genotypes and treatments. **(D)** *Kras^G12D/+^;Trp53*^−/−^ cells are transduced with dox-inducible shRNAs, treated with dox for 72 h, and used for immunoblot (top) and RT-qPCR (bottom) with the indicated antibodies and primers, respectively. **(E)** Combined dox and GSK343/GSK669 treatment followed by immunoblot (top). The colony-formation assay for the indicated genotypes is performed under the same experimental conditions as in A and B (lower panel). (**F)**. Cells were infected with a dox-inducible EZH2 shRNA and treated (or not) with dox and GSK343 inhibitor. After 3 d, cells were treated 45 min with BrdU (10 μM), and FACS analysis was performed. One representative cell cycle profile for the indicated cell line is shown. Error bars represent SD. P values were calculated using Student’s *t* test. *, P ≤ 0.05; **, P ≤ 0.01; ***, P ≤ 0.001. Data are based on three independent experiments.

To investigate the role of Ezh2 in *Kras^G12D/+^* NSCLC cells, we established primary cultures from mouse *Kras^G12D/+^* and *Kras^G12D/+^;Trp53*^−/−^ lung adenocarcinoma tumors. Applying the Ezh2-specific inhibitor GSK343 resulted in a down-regulation of H3K27me3 and led to reduced cell proliferation and colony formation as compared with cultures treated with an inactive control compound. This occurred in both genotypes (Fig. 1, A–C). Similar results were obtained with stable dox-inducible Ezh2 depletion or with transient siRNAs, without affecting the level of expression of other polycomb protein members (Fig. 1 D). To totally deplete activity for Ezh2 in NSCLC cells, we combined simultaneous dox and GSK343 treatment, and this resulted in the synergistic inhibition of cell growth (Fig. 1 E). We attributed this to a clearly measurable arrest of the cell cycle in the G0/G1 phase (Fig. 1 F).

**Figure 2.**
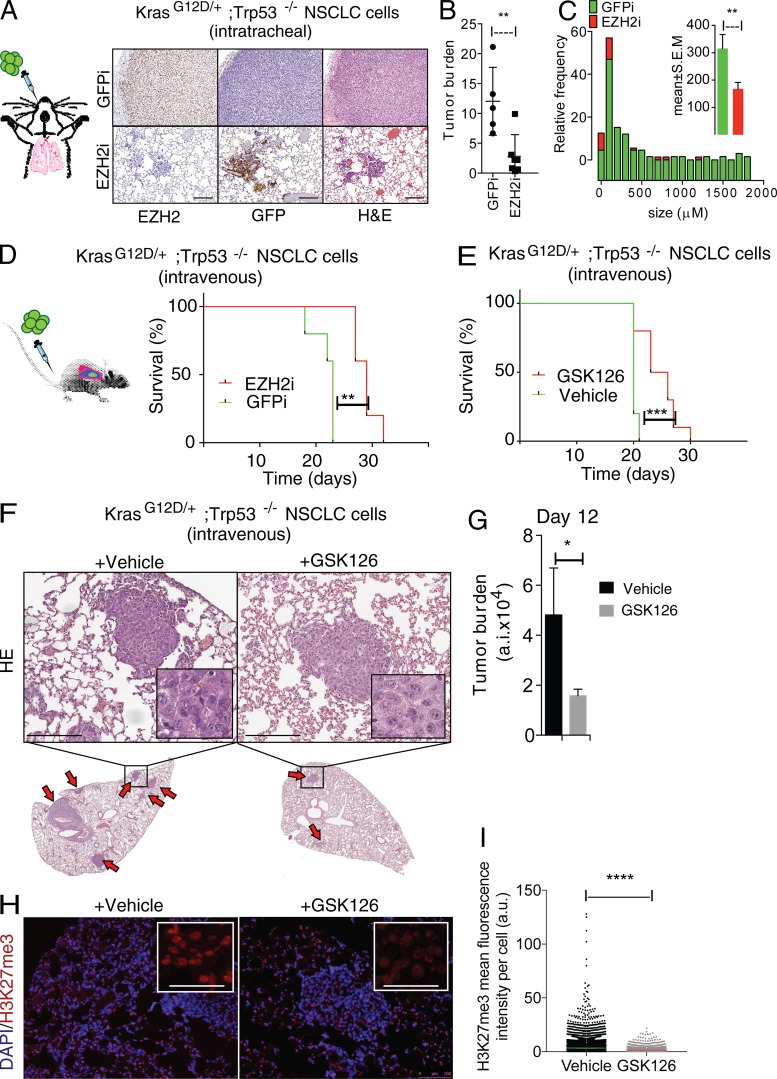
**Ezh2 is homogeneously expressed in Kras-driven NSCLC grafts and its inhibition impairs tumor growth in vivo. (A)** Ezh2, GFP IHC, and H&E stainings of lung adenoma formation after intratracheal transplantation (left) for dox-inducible *Ezh2* knockdown (Ezh2i; bottom) compared with controls (GFPi; top). **(B and C)** ImageJ quantification of the tumor burden and frequency distribution of lesions. **(D)** Overall survival (OS) of mice intravenously transplanted with *Kras^G12D/+^;Trp53*^−/−^;Ezh2i (*n* = 5) or *Kras^G12D/+^;Trp53*^−/−^;GFPi cells (*n* = 5) and treated with dox drinking water (P < 0.004). **(E)** Overall survival of mice intravenously transplanted with *Kras^G12D/+^;Trp53*^−/−^ cells and treated daily with 50 mg/kg GSK126 (*n* = 10) or vehicle (*n* = 10, right; P *<* 0.0003; see also Fig. S1). P values are by log-rank (survival). **(F)** Representative H&E staining of day 12 lung sections from a time-course experiment of mice intravenously transplanted with *Kras^G12D/+^;Trp53*^−/−^ cells and treated daily with 50 mg/kg GSK126 (*n* = 4) or vehicle (*n* = 3). Similar-sized lesions are magnified to show the differential cytoplasmic staining. Arrows denote lesions. **(G)** ImageJ quantification of the tumor burden. a.i., arbitrary intensity. **(H)** Representative immunofluorescence for GSK126 target H3K27me3 and DAPI performed in the same groups as in F. **(I)** ImageJ quantification of the anti-H3K27me3 intensity. a.u., arbitrary units. Five fields were counted per tumor. *, P ≤ 0.05; **, P ≤ 0.01; ***, P ≤ 0.001; ****, P < 0.0001 versus vehicle by unpaired *t* test. Error bars represent mean ± SEM. Scale bars represent 100 µm.

To test whether Ezh2 inhibition affected tumor cell proliferation in vivo, we performed orthotopic transplantation experiments. We intratracheally injected the most aggressive *Kras^G12D/+^;Trp53*^−/−^ cells expressing a dox-inducible short hairpin to knock down Ezh2 (Ezh2i) or GFP (GFPi) as control. The NSCLC cells were also transduced with luciferase to permit longitudinal assessments of grafting success and tumor progression. The tumors that developed in these orthotopic models homogeneously expressed Ezh2 at high levels (Fig. 2 A). We continuously administered dox through the drinking water, leading to the depletion of Ezh2, followed by a significant impairment of the formation of adenomas compared with the GFPi controls (Fig. 2 B). At the selected time point, the lung lesions of mice that received the Ezh2i cells were significantly smaller; they generally scored as mild hyperplasia and rarely progressed to severe hyperplasia or adenoma in contrast to the GFPi controls (Fig. 2 C; data not shown).

Next, we turned to tail vein grafting and used noninvasive bioluminescence. In contrast to the intratracheal procedure, via tail vein injection, efficient lung colonization occurred before genetic depletion, and animals bearing Ezh2-depleted tumors developed lung cancer, possibly because of reduced impact of grafting stress. Yet, a moderate and significant increase in the lifespan of the Ezh2i group was observed compared with GFPi control mice (Fig. 2 D). Similar results were obtained when depleting Eed, another component of the PRC2 complex (see below). Most importantly, a pharmacological approach based on daily dosing of GSK126, a clinically relevant Ezh2 inhibitor, also promoted a moderate and significant tumor growth inhibition and extension in survival (Fig. 2 E). Luciferase imaging was performed at day 2 (when lung colonization is complete) and again 20 d after treatment (when control animals display shortness of breath). The comparison indicated that the growth of tumors is reduced in GSK126-treated mice (data not shown). In a time-course experiment, histological analysis revealed that lung lesions exposed to GSK126 for 10 d are on average significantly smaller and display a pale cytoplasm compared with vehicle-treated ones (Fig. 2, F and G). To confirm that the in vivo effects of GSK126 on NSCLC progression are on target, we performed immunohistochemistry (IHC) to detect histone H3K27 tri-methylation, the enzymatic product of Ezh2, and found significant depletion of this histone mark in the treated tumors compared with controls (Fig. 2, H and I).

These results suggest that the function of PRC2 is important for the proliferation of *Kras^G12D^*-driven NSCLC cells. In addition, orthotopic transplantation of these cells represents a practical in vivo model for studying treatment response and development of resistance to therapeutic intervention in lung lesions expressing Ezh2.

### Ezh2 inhibition promotes the transcriptional amplification of an inflammatory program involving NF-κB and dampens EGFR/ERBB2 signaling

To investigate the mechanisms behind the cellular responses to PRC2 inhibition in vivo, we performed genome-wide expression profiling of freshly isolated cells from tumors derived from intravenous transplantations. The different methods to inhibit Ezh2 (treatment with GSK126 or the administration of dox) allowed us to distinguish between effects that were the direct result of EZH2 inhibition versus off-target effects due to the specific treatment.

**Figure 3.**
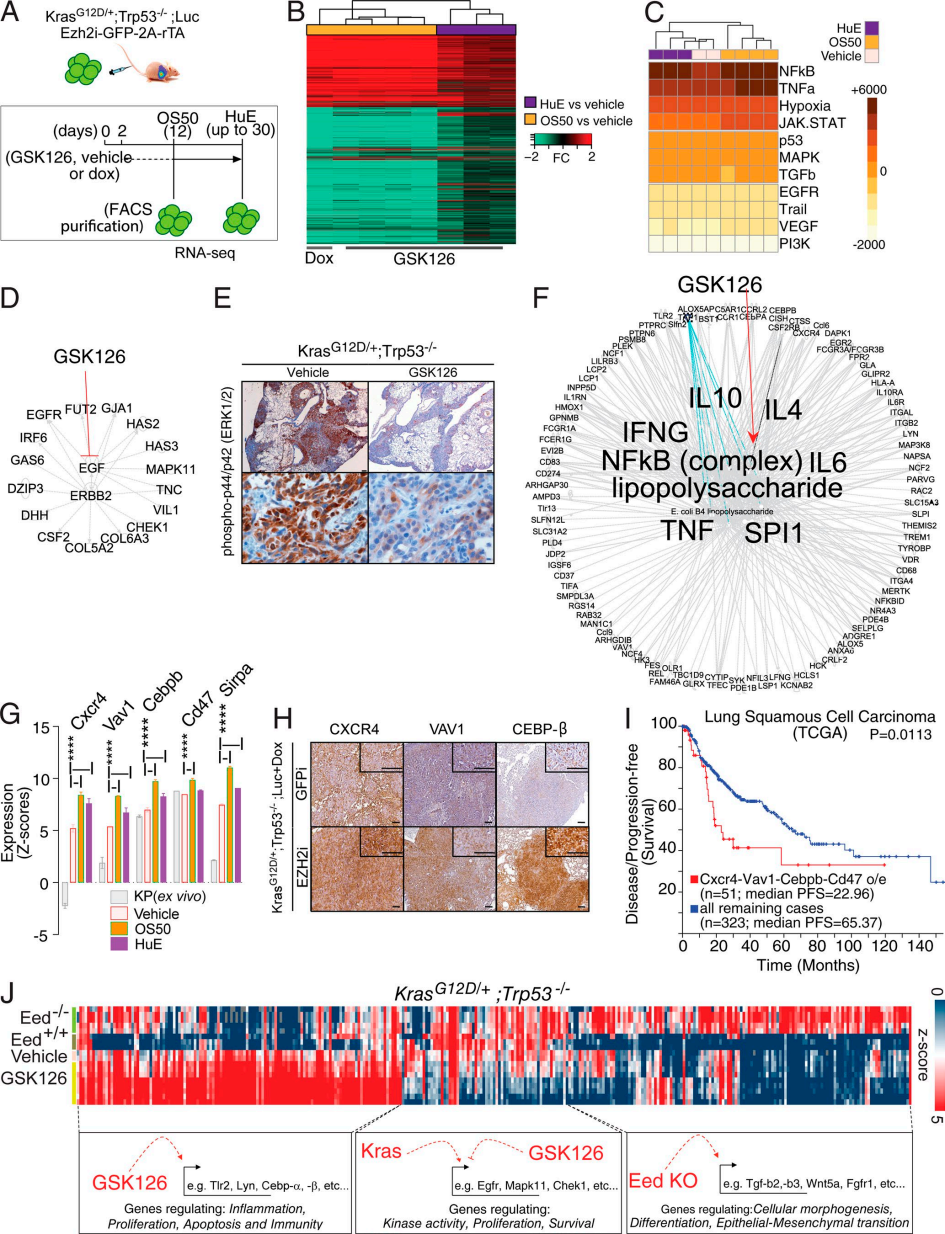
**Ezh2 inhibition promotes the transcriptional amplification of an inflammatory program at the expense of EGFR/ERBB2 signaling. (A)** Outline for gene expression profiling of GSK126- or dox-mediated Ezh2 inhibition in *Kras^G12D/+^;Trp53*^−/−^;Ezh2-2A-rTA;Luc lung tumors in vivo upon FACS purification followed by RNA-seq. **(B)** Heat map illustrating up- and down-regulated genes in animals treated with GSK126 for 10 consecutive days (OS50; *n* = 4) or until HuE with GSK126 (*n* = 3) or dox (*n* = 1). Fold changes (FC) refer to vehicle-treated animals (*n* = 2). **(C)** Heat map illustrating pathway activation inferred by PROGENy scores (color) associated with RNA-seq data for GSK126- and vehicle treated animals. Unsupervised hierarchical clustering was based on Manhattan distance. **(D)** IPA for upstream regulators of genes down-regulated by PRC2 inhibition (counts per million in the vehicle >7, FC < −1.1 in OS50 and HuE). Note the concordance with NF-κB/TNF-α activation by PROGENy. **(E)** Representative IHC for p-p44/42 in lung tumors with GSK126 or vehicle as validation for EGFR signaling down-regulation as in shown in D. **(F)** As in D, IPA for upstream regulators of genes induced by PRC2 inhibition in all conditions (*n* = 212, FC > 1.1). **(G)** Selected expression data for potential oncogenes and negative regulators of the antitumor immune response. ****, P ≤ 0.0001. P values were calculated by two-way ANOVA and Dunnett post-hoc test. **(H)** IHC validation of GSK126 up-regulated genes using independent in vivo PRC2 inhibition through dox-mediated Ezh2 ablation. GFP knockdown provided control for shRNA activity. **(I)** Correlation between disease-free survival and overall survival for patients with high or normal levels expression levels for the indicated genes in the TCGA NSCLC database. Data are from http://www.cbioportal.org/, and the P value was calculated using log-rank analysis. **(J)** Heat map illustrating expression (color) for genes differentially regulated in GSK126- and vehicle-treated animals as well as in tumor cells freshly derived from autochthonous lung tumors with the indicated genotypes (DESeq2, P < 0.05). Unsupervised hierarchical clustering for genes was based on Pearson correlation. Note the IPA for pathways associated with individual clusters and the concordance with C, D, and F. Scale bars represent 100 µM.

As the effects of Ezh2 inhibition in the tumors could be dependent on genes directly regulated by PRC2 and on secondary effects, we purified tumor cells at two time points to capture both types of data. The first was at 12 d after the introduction of tumor cells, an asymptomatic stage in which Ezh2 depletion is tumor suppressive (Fig. 2, F and G) and representing half of the median survival time in control animals (hereafter referred to as OS50). The second time point was the individual onset of severe shortness of breath (up to 30 d; hereafter referred to as humane endpoint [HuE]). To yield an enrichment of tumor cells, we isolated GFP-expressing tumor cells by FACS (Fig. 3 A).

To characterize the transcriptional response, we compared tumor cells derived from vehicle/GSK126 with dox-treated orthotopic models and identified differentially regulated genes (DRGs). A normalization of the RNA sequencing (RNA-seq) reads over vehicle-treated controls revealed a striking number of DRGs in tumor cells treated with Ezh2 inhibitors (Fig. 3 B). To identify differences reflecting changes in signaling pathways, we used a method called PROGENy. This method analyzes gene expression data and ranks patterns observed in activated pathways by consulting a curated database that includes the effects of human cancer driver mutations and drug perturbations (Schubert et al., 2018). The pathways most highly activated by GSK126, according to this analysis, were NF-κB and its upstream regulator, TNF-α, followed by the JAK/STAT pathway, which is activated, among others, by IL-6 (Fig. 3 C).

Next, we used Ingenuity Pathway Analysis (IPA) to capture the information of both activated and inactivated pathways. In the latter category, we found a connection to the extended survival of GSK126-treated animals, because several of the genes that were down-regulated by the treatment played a role in tumor cell proliferation, such as those in the EGFR/ERBB2 signaling pathway (Fig. 3 D). We independently validated this finding by probing GSK126-treated and controls for ERK1/2 phosphorylation, which acts as a molecular beacon downstream in this signaling cascade (Fig. 3 E).

To identify genes that were potentially being directly repressed by PRC2, we analyzed all the genes that underwent up-regulation compared with controls. The results of IPA confirmed and extended the outcome from PROGENy; the majority of these up-regulated genes belonged to inflammation-related pathways (113/212 [53%]; Fig. 3 F). The amplified inflammatory signature includes TNF, LPS, IFN-γ, and IL-6, which reinforces the prediction that the NF-κB transcriptional network is being activated (Fig. 3 F). The gene list includes potential oncogenes such as CXCR4, VAV1, and CEBP-β, alongside critical targets such as CD47 and SIRPA that modulate immunosurveillance and are targets for immunotherapy (Fig. 3 G). IHC in independent tumors in which Ezh2 had been down-regulated by dox (*Kras^G12D/+^;Trp53*^−/−^;Ezh2i+dox) confirmed that the Ezh2 loss is specifically associated with an up-regulation of these proteins in vivo (Fig. 3 H). To understand the relevance of these oncogenes in the progression of human NSCLC, we queried a large dataset from The Cancer Genome Atlas (TCGA; http://www.cbioportal.org). Given that these targets were discovered mainly as PRC2 target in lung adenocarcinoma and thereby lowly expressed in this malignancy, we tested their prognostic significance as oncogenes in a different subtype of lung cancer. In lung squamous carcinoma patients, the elevated expression of CXCR4, VAV1, CEBP, and CD47 was correlated with poor patient prognosis (Fig. 3 I).

As we have recently shown that loss of the PRC2 gatekeeper Eed promotes inflammation and epithelial-to-mesenchymal transition (EMT) in autochthonous models of Kras-driven NSCLC (Serresi et al., 2016), we next wished to understand whether pharmacological Ezh2 inhibition phenocopies Eed deletion in full. To this end, we compared RNA-seq on in vivo GSK126-treated *Kras^G12D/+^;Trp53*^−/−^ and controls to those generated by limited ex vivo propagation of *Kras^G12D/+^;Trp53*^−/−^ and *Kras^G12D/+^;Trp53*^−/−^*;Eed*^−/−^ tumor cells. We subjected the RNA-seq profiles to unbiased principal component analysis (PCA) and clustering based on Pearson correlation. These analyses confirmed that in GSK126-treated tumors, the transcriptional amplification of genes related to acute inflammation seemed to be specifically associated with the in vivo enzymatic inhibition of Ezh2 by GSK126 (Fig. 3 J; data not shown). In contrast, genes involved in cellular morphogenesis and EMT remained associated only with cells in which Kras tumorigenesis occurred in the absence of PRC2 as an outcome of Eed deletion in nontransformed cells.

Overall, this analysis indicates that in vivo PRC2 inhibition has milder consequences than PRC2 deletion during tumor initiation in mouse models of Kras-driven NSCLC (Serresi et al., 2016). This means that the Ezh2 inhibition triggers the transcriptional amplification of a program related to inflammation, but not to EMT.

### Transcriptional amplification by Ezh2 inhibition occurs at PRC2-regulated chromatin

**Figure 4.**
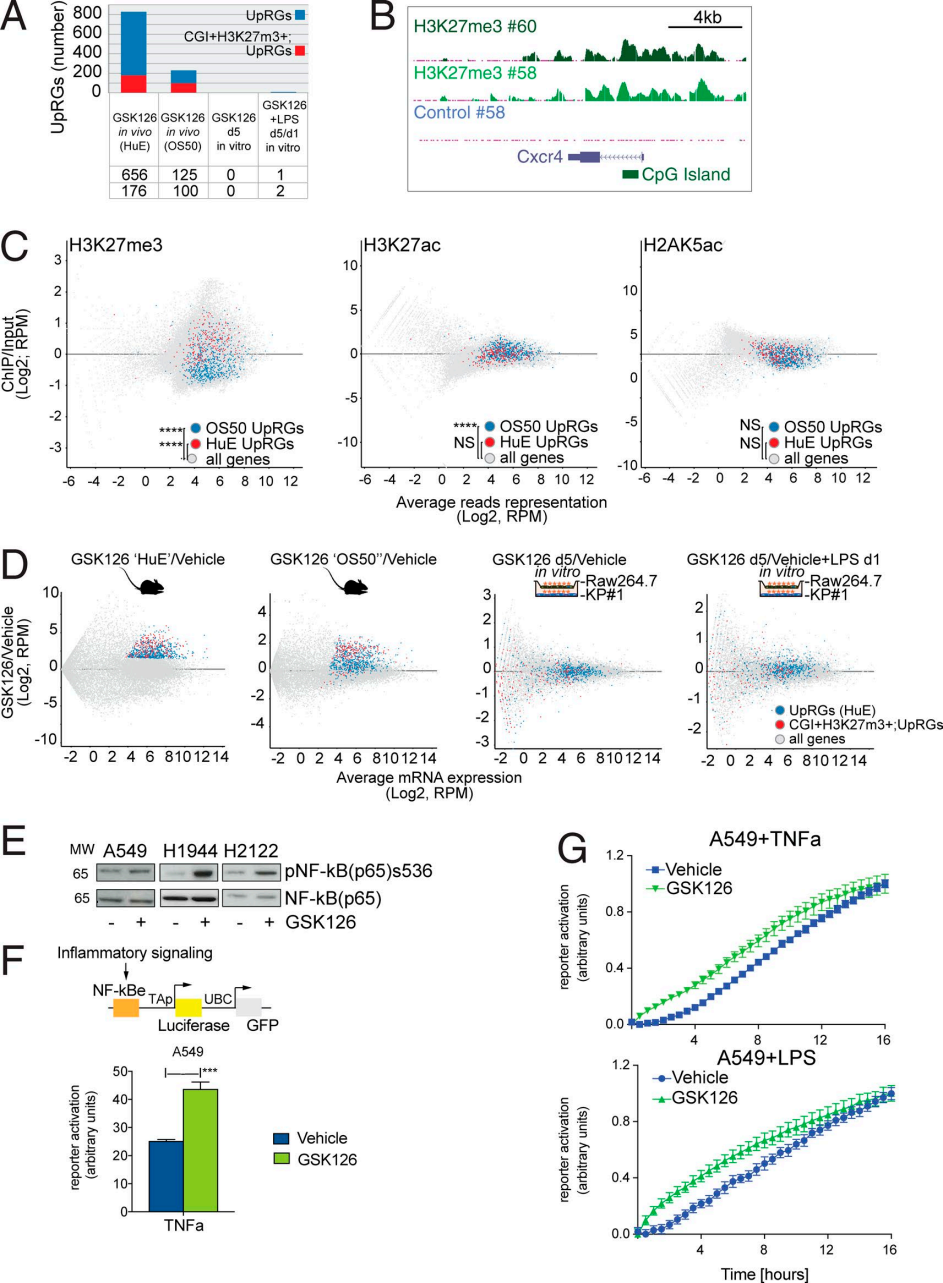
**Transcriptional amplification by Ezh2 inhibition involves PRC2 target chromatin and reinforces NF-κB transcription. (A)** Overlap between genes up-regulated by GSK126 in vivo (blue) and genes decorated with H3K27me3 at CGI in vivo (red) within the indicated transcriptomes. UpRGs, up-regulated genes. **(B)** UCSC genome browser example of H3K27me3 on the Cxcr4 gene locus in two selected *Kras^G12D/+^;Trp53*^−/−^;Ezh2-2A-rTA;Luc lung tumors in vivo. **(C)** Gene set intensity difference analysis for the indicated gene sets in in vivo PRC2-target chromatin (H3K27me3, *n* = 4; H3K27ac, *n* = 2) and control (H2AK5, *n* = 2) over control (input, *n* = 2). Note that initial response to GSK126 (OS50) involves genes decorated by either H3K27me3 or H3K27ac, but not H2AK5ac. RPM, reads per million. **(D)** MA plot for differential gene expression profiling of GSK126 (HuE, *n* = 3; OS50, *n* = 4) or vehicle-treated *Kras^G12D/+^;Trp53*^−/−^;Ezh2-2A-rTA;Luc lung tumors in vivo or in vitro co-cultured with the macrophage cell line Raw264.7. Fold change refers to vehicle-treated controls (*n* = 2). Quantification of up-regulated (blue) and CGI H3K27me3 target genes (red) is limited to the in vivo gene expression (HuE) and chromatin profiles. **(E)** Immunoblot for the indicated antibodies and human NSCLC cell lines upon 5 d of continuous GSK126 treatment and low-confluence replating. MW, molecular weight. **(F)** Time point experiment showing response to TNF-α in GSK126-treated and control A549 cells as gauged by an NF-κB reporter assay. ***, P < 0.001 versus vehicle by unpaired *t* test. Genetic tracing reporter is shown (top). UBC, ubiquitin C promoter. NF-κBe, NF-κB responsive element; TAP, minimal TA promoter. **(G)** Longitudinal response to TNF-α and LPS in GSK126-treated and control A549 cells. The interaction between time points and treatment is significant by two-way ANOVA (P < 0.0001). Data are representative of three independent experiments. Error bars represent mean ± SEM.

To gain insights into the mechanistic implications of NSCLC response to Ezh2 inhibition in vivo, we next investigated whether GSK126-regulated genes involve direct PRC2 targets by interrogating a chromatin immunoprecipitation sequencing (ChIP-seq) profile of H3K27me3 previously generated in *Kras^G12D/+^;Trp53*^−/−^ orthotopic lung tumors (Serresi et al., 2016). While prominent GSK126-up-regulated genes such as *Cxcr4* are indeed decorated with H3K27me3 at CpG islands (CGI), a sizeable fraction of DRGs do not belong to the category of classic PRC2 targets (Fig. 4, A and B).

To test whether the transcriptional amplification involved pre-activated chromatin, we generated ChIP-seq profiling for H3K27ac and H2AK5ac in *Kras^G12D/+^;Trp53*^−/−^ orthotopic lung tumors. Systematically comparing all chromatin profiles with input controls using gene set enrichment analysis revealed that GSK126–up-regulated genes were located in chromatin significantly decorated with either H3K27me3 or H3K27ac, but not H2AK5ac (Fig. 4 C).

To investigate whether the inhibition of Ezh2 is sufficient to promote the transcriptional activation of the specific set of GSK126-regulated genes in tumor cells or additional signaling is required, we generated RNA-seq profiles of *Kras^G12D^;Trp53*^−/−^ NSCLC cells in vitro and compared these profiles to those from the in vivo setting. To account for non–cell-autonomous factors, we co-cultured tumor cells with the macrophage cell line Raw267.4 in the presence of short-term Ezh2 inhibition and further mimicked proinflammatory stimulation using LPS. Consistent with data in Fig. 3 J and the well-known context-dependent function attributed to PRC2, comparative analyses indicated that up-regulation of in vivo GSK126-regulated genes (see Materials and methods) is specific for the orthotopic growth setting, whereas in vitro Ezh2 inhibition affected the expression of a different set of genes (Fig. 4 D).

Since the TNF-α/NF-κB axis was prominently activated in the in vivo gene expression profile of GSK126-treated animals, we tested whether GSK126 can directly activate the NF-κB pathway or reinforce its activation by other pathways. To reach a broad conclusion on this matter, we performed this experiment in human Kras-driven NSCLC cell lines. Three lines were propagated in the presence of GSK126 or vehicle, and we searched for evidence of NF-κB pathway activation by p65 phosphorylation. Notably, p65 was phosphorylated in a GSK126-dependent manner in three different cell lines, but only when reseeded at low confluence, thereby indicating that Ezh2 inhibition enhances NF-κB activation in response to stress (Fig. 4 E). Since a similar activation of p65 is observed in the three human NSCLC cell lines, we next selected A549 cells for subsequent experiments and exposed these for 3 h to the NF-κB upstream activator TNF-α and measured NF-κB transcriptional activation using a luciferase reporter (Wilson et al., 2013). This experiment revealed that GSK126 treatment enhanced NF-κB activity in response to TNF-α stimulation (Fig. 4 F). Importantly, in a continuous stimulation of 16 h by the NF-κB activators TNF-α and LPS, the transcriptional activity of GSK126-treated cells reached a plateau more swiftly than control cells (Fig. 4 G).

Thus, our results suggest that Ezh2 inhibition in vivo cooperated with inflammatory signaling and facilitates the transcriptional amplification of proinflammatory genes residing in PRC2-regulated chromatin.

### In vivo Ezh2 inhibition affects the tumor microenvironment

**Figure 5.**
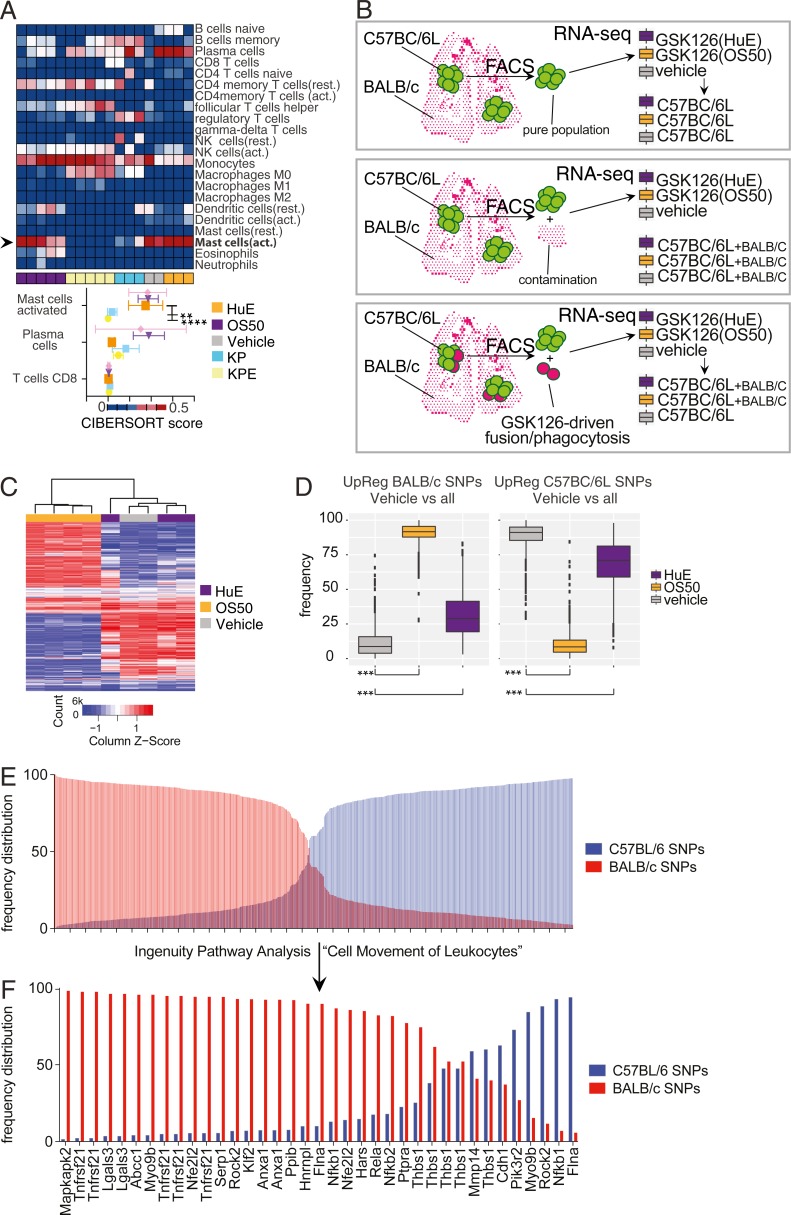
**In vivo Ezh2 inhibition affects the tumor microenvironment. (A)** Heat map illustrating potential immune cells infiltrating the orthotopic lung tumors and detected in RNA-seq (Fig. 3 B) by cell-type identification by estimating relative subsets of RNA transcripts (CIBERSORT). Quantification of the in vivo–specific populations (HuE, *n* = 3; OS50, *n* = 4; vehicle-treated controls, *n* = 2) as gauged by two-way ANOVA and Dunnett’s post test (bottom). ****, P < 0.0001; **, P < 0.01. Rest., resting; act., activated. **(B)** Models depicting potential sources of non–tumor cell–derived reads in tumor-enriched RNA-seq. **(C and D)** Heat map and box plot illustrating SNP quantification from RNA-seq data in Fig. 3 B without a reference genome using KisSplice. Note the (host-derived) BALB/c reads are significantly enriched at an early time point for in vivo GSK126 treatment and subsequently depleted compared with tumor-derived C57BC/6L reads. ***, P < 0.001 by one-way ANOVA. **(E and F)** Bar chart quantification of genes bearing BALB/c or C57BC/6L SNPs. Shown are all genes (top) and those restricted to the IPA category “Cell Movement of Leukocytes” (bottom).

To understand whether Ezh2 inhibition or the observed inflammatory activation may result in non–cell-autonomous effects, we subjected our gene profiling experiment to a bioinformatic method by which the immune cell composition within a tissue can be inferred (Newman et al., 2015). Despite the fact that our RNA-seq profiles were enriched for tumor cells, in silico prediction by CIBERSORT (Newman et al., 2015) revealed that orthotopic lung cancer grafts present indirect evidence for infiltration by activated mast cells (Fig. 5 A). The mast cell signature was discovered only within the gene expression profile of freshly derived and not ex-vivo–propagated tumor cells. Moreover, the orthotopic tumors generated in nude mice had no traces of adaptive immune effectors such as CD8 T cells, thereby supporting the specificity of this analysis (Fig. 5 A, bottom).

Tumor homeostasis includes infiltration by non–tumor cells or events of fusion or phagocytosis between host and donor cells. As the mast cell activation signature is shared between GSK126-treated and naive tumors, the presence of donor- and recipient-derived RNA molecules offered the opportunity to investigate if any of these events could be specifically attributed to GSK126 (Fig. 5 B). To this end, we applied a method for detecting single-nucleotide polymorphisms (SNPs) in RNA-seq reads independently of a reference genome (Lopez-Maestre et al., 2016). Strikingly, KisSplice revealed that host-derived BALB/c-specific SNPs were selectively enriched in reads from the GSK126-treated cohort, with a spike representing a significant contribution from the host upon ten days of Ezh2 inhibition, which later fell (P < 0.001; Fig. 5, C and D). The specificity of this analysis was confirmed by the fact that donor-specific SNPs underwent an opposite regulation (P < 0.001; Fig. 5, C and D), as well as by the fact that some genes could be largely attributed to either genetic background, which suggests the host-derived reads may be contributed by a homogeneous cell population (Fig. 5 E). In line with CIBERSORT, unbiased IPA analysis of genes uniquely identified by the BALB/c SNPs pointed at a leukocyte origin for host-derived reads (Fig. 5 F).

Overall, these data suggest that the in vivo inhibition of Ezh2 in animals with lung cancer alters the tissue microenvironment.

### In vivo RNA interference (RNAi) screening for genes antagonistically regulated by PRC2 and NF-κB uncovers targets with pro-oncogenic potential

We next investigated whether inflammatory signaling helps bypassing the growth defect observed in vitro and in vivo in NSCLC cells in which PRC2 is inhibited.

First, we investigated the interaction between PRC2 loss and the cytokines that stimulate acute inflammation via TLR and TNF signaling in vitro. To this end, we exposed *Kras^G12D/+^;Trp53*^−/−^ cells to dox-mediated PRC2 inhibition with or without LPS plus TNF-α before assessing their colony-formation potential. Notably, the negative effect of PRC2 loss on colony formation was rescued by inflammatory signaling (Fig. S1).

**Figure 6.**
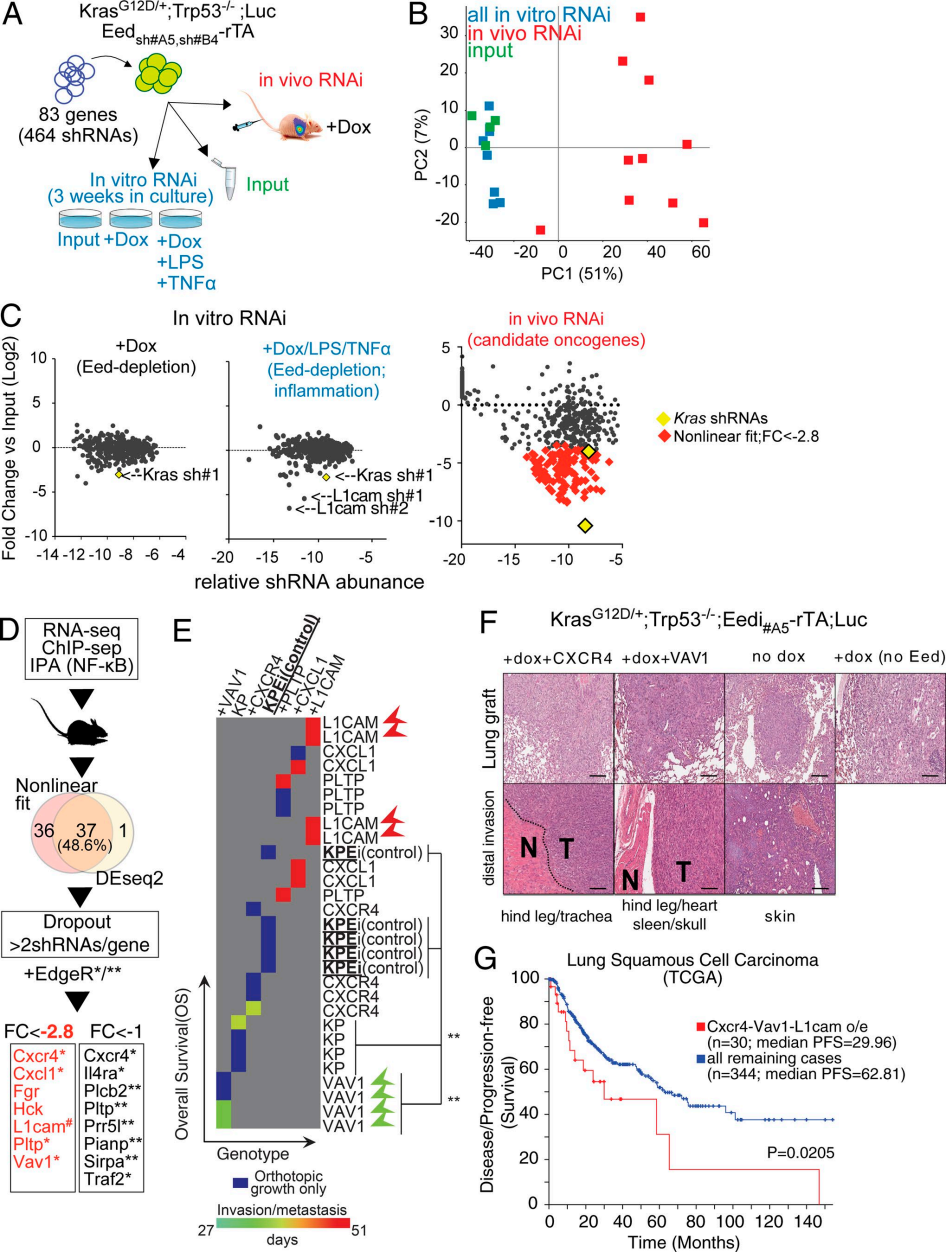
**In vivo RNAi screening for PRC2-repressed inflammatory mediators with pro-oncogenic potential. (A)** Outline of the parallel in vivo/in vitro RNAi screen. **(B)** PCA RNAi screen results. Note the clustering of the in vitro samples and inputs (PC1) as well as the minor variation between individual animals (PC2; *n* = 8, 6, and 10, respectively). **(C)** Normalized representation of shRNA segregation. The x axis represents individual hairpin relative abundance normalized by total reads count, and the y axis shows fold-change versus input control. The effect of dox-mediated Eed depletion in vitro is compared with input to combined dox/LPS/TNF-α treatment and to in vivo proliferation. Each dot represents the mean of two biological and two technical replicas (left, middle, and right panels, respectively). The *Kras* sh#1 dropout control and the newly discovered *L1cam* sh#1 and sh#2 are shown. In the right panel, in vivo dropout outliers were obtained by one analysis method and are depicted as red diamond signs. Two independent shRNAs targeting Eed were used in vivo (each *n* = 5). **(D)** Schematic representation of target selection and filtering out of potential off targets. Three analysis methods were used. Asterisks denote genes identified by non-linear fit/P < 0.05 EdgeR (*) and/or DESeq2/ P < 0.05 EdgeR (**). FC, fold change. **(E)** Heat map representation of overall survival and invasion/metastasis data. Each animal (column) received 5 × 10^5^ *Kras^G12D/+^;Trp53*^−/−^;Eedi cells (“KPEi”) intravenously and was treated with dox via drinking water (except for “KP” controls). Groups are different in the overexpression of individual genes as indicated compared with an empty pLKO.1 vector (*n* = 4). Invasion and metastasis were located using bioluminescence and confirmed through histological analysis. Note that dox-induced Eed loss shows no metastasis (box), while VAV1 gain strongly impairs survival and enhances the metastatic phenotype (arrowheads); L1CAM gain enhances the metastatic phenotype while extending survival. P values were calculated using the log-rank (Mantel–Cox) test. **(F)** H&E staining of representative primary tumors and bioluminescence-detected sites of distal invasion. *Kras^G12D/+^;Trp53*^−/−^;Eed_#a5_-2A-rTA;Luc (modified as indicated) were intravenously injected into nude mice, which were sacrificed at the onset of shortness of breath and/or metastasis. Scale bars represent 100 µM. N, normal; T, tumor. **(G)** Correlation between disease-free survival and overall survival for patients with high or normal levels expression levels for the indicated genes in the TCGA NSCLC database. PFS, progression-free survival. Data are from http://www.cbioportal.org/, and P value was calculated using log-rank analysis.

Next, we exploited a ChIP-seq–instructed orthotopic in vivo shRNA screening approach (Gargiulo et al., 2013, 2014). This enables screening for potential oncogenes antagonistically regulated by PRC2 and inflammation in vivo. For this screen, we selected genes that are directly repressed by Ezh2, up-regulated by GSK126, and regulated by NF-κB according to IPA. We reasoned that this set of 83 genes (hereafter “PRC2-inflammatory mediators”) may be enriched for relevant oncogenes driving tumor progression upon Ezh2 inhibition by GSK126. With these premises, we performed a parallel in vivo/in vitro screen to systematically interrogate the potential regulators of tumor growth. Individual silencing in *Kras^G12D/+^;Trp53*^−/−^ cells was achieved through a pool of 464 shRNAs. Statistical representation of this library exceeded 2,000-fold, which is sufficiently large to compensate for stochastic dropout during grafting (Gargiulo et al., 2014). As a functional readout to determine significant hits for in vivo dropouts, we included two validated anti-*Kras* shRNAs as controls (Fig. 6 A). To mechanistically ensure PRC2 depletion at target genes and perform the parallel screening procedure under optimal conditions, we chose to inhibit PRC2 using a dox-mediated ablation of Eed. Eed depletion confers comparable in vivo survival advantage (see below) but is a more potent growth inhibitor in vitro compared with GSK126 and Ezh2 depletion. In vivo, we used ten animals divided into two pools of five, each carrying NSCLC cells transduced at low multiplicity of infection with the library and with either one of two independent dox-inducible shRNAs (Eedi). In parallel, we compared tumor cell growth in vitro under constant dox-mediated Eed depletion alone or in combination with LPS plus TNF-α. Importantly, the T0 control samples and the in vitro samples showed a substantial similarity in PCA and segregated away from the in vivo samples. Moreover, by comparing the average hairpin counts for the two groups with the different Eed shRNAs, PRC2-inflammatory mediators appeared reproducibly enriched or depleted (*R*^2^ = 0.8; Fig. 6 B; data not shown). Overall, the quality of the screen met the criteria for interrogating dropout shRNAs as a proxy for pro-oncogenic functions.

Interestingly, 113 out of 430 shRNAs scored as dropouts with 2.8 < X < 10.4–fold changes in lung tumors generated under constant dox-treatment (Fig. 6 C). In vitro, the same approach returned only Kras and L1cam as dropouts (Fig. 6 C). In vivo, our selection based on the 2.8 < X < 10.4–fold change window is stringent enough to exclude the least effective Kras hairpin and, as such, it critically implicates the remaining PRC2-inflammatory mediators in oncogenesis.

To increase the stringency of our analysis further and reduce the incidence of off-target effects, we applied a method based on linear regression and outlier identification and called as hits seven genes that were identified by more than two shRNAs. Independently, we applied two other raw read count–based statistical methods, EdgeR and DESeq2 (Fig. 6 D). We used in vivo genetic complementation as an orthogonal validation of the in vivo RNAi screen. We individually overexpressed hits from the less conservative method, such as Vav1, Cxcr4, Pltp, Cxcl1, and L1cam in *Eed*-depleted *Kras^G12D/+^;Trp53*^−/−^ cells, and used these in orthotopic transplantations. Eed depletion conferred significant survival extension to the NSCLC-bearing animals (Fig. 6 E), which indicates that PRC2 inhibition in vivo using dox-inducible Eedi is comparable to Ezh2i and GSK126 (Fig. 2, D and E). Strikingly, when overexpressing individual genes identified as “dropout” in our screen, we observed the acquisition of an invasion/metastasis phenotype, a feature that is extremely rare in *Kras^G12D/+^;Trp53*^−/−^ orthotopic tumors and absent in *Eed*-depleted *Kras^G12D/+^;Trp53*^−/−^ controls (Fig. 6, E and F).

Although full comparisons between individual genes are complicated by potential differences in their levels of overexpression, this experiment revealed two distinct types of oncogenes. VAV1 and, to a more limited extent, CXCR4 both imparted prometastatic activity and also decreased overall survival, but only in vivo. Otherwise, L1CAM appeared to be required for growth in vitro under inflammatory signaling, while its in vivo overexpression marked an increase in the prometastatic potential of NSCLC, despite the fact that this also appeared to extend the overall survival (Fig. 6, E and F). Consistent with predictions based on our experimental data, correlative analyses showed that NSCLC progresses faster in TCGA patients with an elevated expression of CXCR4, VAV1, and L1CAM (Fig. 6 G).

Collectively, our data indicate that the transcriptional program that is amplified in vivo through the inhibition of PRC2 in NSCLC includes genes that are (1) downstream proinflammatory signaling, (2) pro-oncogenic, and (3) prometastatic.

### Ezh2 inhibition enhances responses to BTZ and NM in Kras NSCLC grafts

To determine whether the amplification of an inflammatory program by GSK126 could be therapeutically exploited in NSCLC, we next investigated whether applying GSK126 would enhance responses when cells were subsequently treated with a combination of BTZ plus NM, which target the proteasome-dependent activation of NF-κB, and inflammation induced by Kras and Ezh2 depletion, respectively.

**Figure 7.**
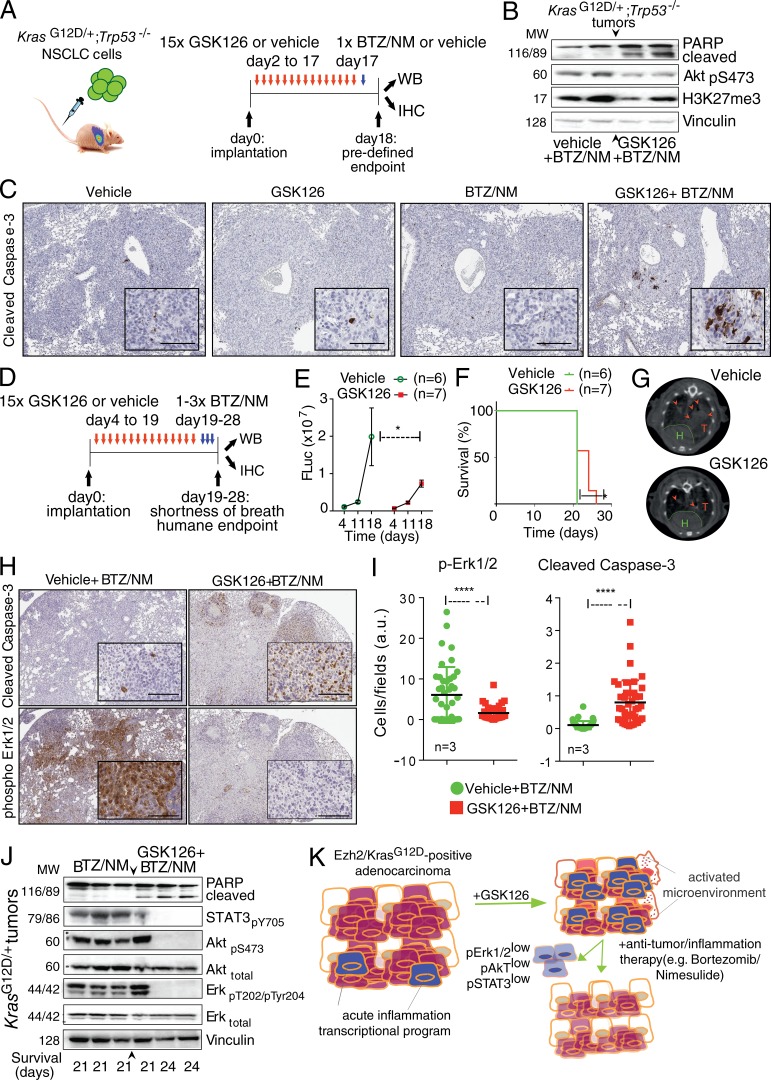
**Ezh2 inhibition enhances response to BTZ/NM in Kras-driven NSCLC grafts. (A)** Outline of in vivo sequential treatment of *Kras^G12D/+^;Trp53*^−/−^ grafts with GSK126/vehicle and MTD of BTZ/NM. WB, Western blot. **(B and C)** Immunoblot and representative IHC (*n* = 3) of *Kras^G12D/+^;Trp53*^−/−^ tumors from A using the indicated antibodies. MW, molecular weight. **(D)** Outline of in vivo sequential treatment of *Kras^G12D/+^;Trp53*^−/−^ grafts with GSK126/vehicle and BTZ/NM. **(E)** Longitudinal imaging of tumor growth for the experiment in (D) by luminescence. P value is by two-way ANOVA. **(F)** Overall survival of mice in E. **(G)** Representative micro-CT scans of *Kras^G12D/+^;Trp53*^−/−^ vehicle-treated tumors and of good responders to in vivo GSK126 (H, heart; T and arrowheads, tumors). **(H)** Representative IHC (*n* = 3) of *Kras^G12D/+^;Trp53*^−/−^ tumors from D and E using the indicated antibodies. **(I)** Quantification of cells showing immunoreactivity to the indicated antibodies. The full lung area for individual animals (*n* = 3) was divided into equal-sized fields, and ten fields were subjected to ImageJ automated counting. a.u., arbitrary units. **(J)** Immunoblot of *Kras^G12D/+^;Trp53*^−/−^ tumors from D and E using the indicated antibodies. **(K)** A model for therapeutic PRC2 inhibition in Kras-driven NSCLC. GSK126 amplifies acute inflammation in Ezh2-expressing NSCLC and modifies the tumor tissue and its microenvironment enhancing the response to subsequent selected treatments such as proteasome inhibitors and anti-inflammatory drugs. Error bars represent mean ± SEM. Scale bars represent 100 µM. *, P ≤ 0.05; ****, P ≤ 0.0001.

Since *Kras^G12D/+^*-driven tumors have been reported to be intrinsically resistant to BTZ or quickly develop adaptive resistance (Xue et al., 2011), we first tested whether inhibiting PRC2 in vivo would enhance responses to a subsequent treatment using the maximum tolerated dose (MTD) of BTZ/NM. Our gene expression profile experiment suggested that 15 d of continuous GSK126 exposure is sufficient to stimulate an adaptive response involving inflammation. To assess the response to each treatment applied individually in vivo, we performed daily GSK126 therapeutic interventions in the *Kras^G12D/+^;Trp53*^−/−^ NSCLC model after verifying tumor take (day 2) and randomizing the animals into two groups (*n* = 10 per group). GSK126 and vehicle mice were further randomized after 15 d to receive BTZ, mimicking a previous preclinical validation of BTZ in this model (Xue et al., 2011). Under these experimental conditions (Fig. 7 A), we delivered a single MTD of BTZ and NM (see Materials and methods). 20 h later, mice were sacrificed and randomized for analysis of proliferation/apoptotic markers by either immunoblotting or IHC. We observed that GSK126 preconditioning resulted in impaired phospho-Akt and induced Parp1 and Caspase-3 cleavage in response to BTZ/NM treatment (Fig. 7, B and C).

Next, we decided to test a dosing schedule that could be applied until the mice reached the HuE. To permit homogenous and efficient tumor grafting, mice were treated with GSK126 or vehicle starting from day 4 after transplantation and randomized. *Kras^G12D/+^;Trp53*^−/−^ tumor-bearing mice with either GSK126 (*n* = 7) or vehicle (*n* = 6) were longitudinally tested for assessing the impact of GSK126 treatment on in vivo tumorigenic potential of orthotopically transplanted cells by luciferase emission. This analysis revealed that at day 18, daily dosages of GSK126 had significantly impaired tumor proliferation compared with vehicle-treated controls, even though the tumors had continued to grow compared with their size at day 4, indicating that the GSK126 treatment was behaving as expected (Fig. 7, D–G). To incorporate a clinically relevant longitudinal imaging of tumor responses to GSK126 in vivo, we also tested the animals using 2D and 3D magnetic resonance imaging (MRI) and micro–computed tomography (micro-CT). The latter detected more clearly good responders among the GSK126 cohort (Fig. 7 G).

At day 19, mice were given doses of BTZ and NM in alternation on successive days (Fig. 7 D; Materials and methods). A severe shortness of breath signaled the HuE for the vehicle → BTZ/NM mice at day 21, while the survival of GSK126 → BTZ/NM was extended (Fig. 7 F). As above, diseased tissues were sorted for either immunoblotting or IHC. Whereas the increased overall survival in the GSK126 cohort may result from the additive effect of the sequential drug treatment, GSK126 significantly promoted the pro-apoptotic response to the BTZ/NM treatment, as measured by cleaved Caspase-3 in tumors of similar size (Fig. 7, H–J), reinforcing the MTD experiment (Fig. 7, A–C). Erk1/2 signaling inhibition was also observed using IHC in these mice, consistent with our data with GSK126 alone (Fig. 7, H and I). Finally, immunoblotting revealed the presence of cleaved Parp1 and the inhibition of Erk1/2 and Akt phosphorylation in the mice that received full cycles of BTZ/NM after GSK126 (Fig. 7 J).

We excluded the possibility that additional genetic alterations were contributing to the animals’ response to BTZ/NM using two independent *Kras^G12D/+^;Trp53*^−/−^ cell lines for in vivo experiments and sequencing their genomic DNA (data not shown).

In summary, our data suggest that the sequential dosing of GSK126 and BTZ/NM affect the proliferation and survival of NSCLC cells in vivo in a process that potentially involves both cell-intrinsic and non–cell-autonomous effects (Fig. 7 K).

### Control of RNA synthesis and proteostasis links Ezh2 inhibition, transcriptional amplification of inflammation, and therapeutic vulnerability

**Figure 8.**
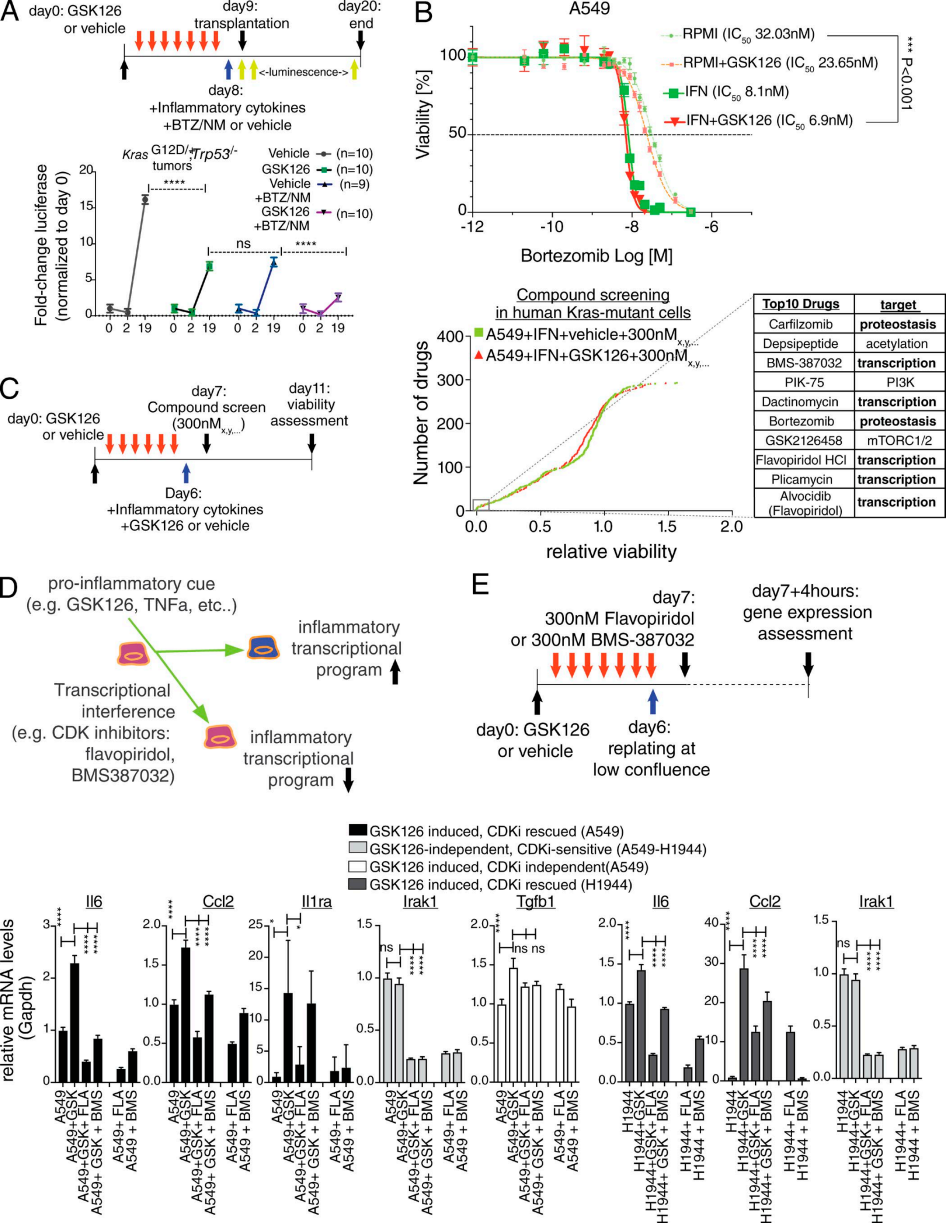
**Control of proteostasis and RNA synthesis links amplification of proinflammatory genes by GSK126 and therapeutic vulnerability. (A)** Schematic outline and tumorigenic potential of *Kras^G12D/+^;Trp53*^−/−^ mouse NSCLC cells upon in vitro sequential dosing of GSK126, BTZ, and NM. Cells were exposed in vitro for 1 wk to 5 µM GSK126 or vehicle followed by 1 d of inflammatory medium (see Materials and methods). Live cells were orthotopically transplanted into nude mice (*n* = 9/10), and luciferase expression was measured at the indicated time points. Note that we used an experimentally derived median inhibitory concentration (IC_50_) for 10 nM BTZ and 50 µM NM because IC_50_ is >600 µM in mouse and human NSCLC cells. Note the synergism in the sequential GSK126/BTZ+NM (all P < 0.0001). P values were calculated by two-way ANOVA. **(B)** Proliferation of A549 cells ± GSK126 propagated either in RPMI + 10% FBS or serum-free medium with inflammatory cytokines (see Materials and methods). Assessment of dose response to BTZ 96 h after treatment. IC_50_ is shown for each condition. Error bars represent mean ± SEM (*n* = 4, biological replica), and P value was calculated by one-way ANOVA and Dunnett’s post-hoc test. **(C)** Schematic outline and compound screen in human Kras-driven NSCLC cells. Cells were treated with GSK126 or vehicle as depicted in the schematic outline until seeded into 384-well plates. After 24 h, the compound library containing 293 unique pharmacologically active compounds was added into the plates. Two concentrations were used (30 nM and 300 nM) in three biological replicates. After 4 d, the medium was changed and supplemented with 1/25th of CellTiter-Blue reagent. After 24 h, the cell viability was measured as fluorescence intensity signals with Envision Multilabel Plate Reader. Cumulative frequency distribution plot shows drugs affecting A549 survival from left to right. The top ten drugs (inset) and their names and target processes are listed on the right. **(D)** A model explaining how control of RNA synthesis is connected to GSK126 amplification of proinflammatory genes. **(E)** Schematic outline and RT-qPCR assessment response for two independent CDK9 inhibitors from the top ten list in B, flavopiridol (FLA) and BMS-387032 (BMS), in human Kras-driven A549 and H1944 NSCLC cells. Error bars represent mean ± SEM (*n* = 3, biological replica), and P value was calculated by two-way ANOVA and Dunnett’s post-hoc test. *, P ≤ 0.05; ****, P ≤ 0.0001. CDK, cycline-dependent kinase; CDKi, cycline-dependent kinase inhibitor.

We next wished to tackle the complex issue of determining the individual responses of lung cancer cells to GSK126 and BTM/NM. To this end, our next efforts aimed to disentangle the in vivo tumorigenic potential of NSCLC from cellular responses to Ezh2 inhibition and inflammation. First, we exposed *Kras^G12D/+^;Trp53*^−/−^ NSCLC cells to GSK126 or BTZ/NM as well as sequential GSK126 → BTZ/NM in vitro*.* We followed this with an in vivo assessment of the tumorigenic potential (Fig. 8 A). Inflammatory signaling stimulation was applied to cells 1 d before transplantation and simultaneously with BTZ/NM treatment in serum-free medium (IFN; see Materials and methods). After a tail vein injection of live cells, all the groups exhibited a similar grafting efficiency. Single treatments with either GSK126 or BTZ showed impaired tumor progression, as did sequential GSK126 → BTZ treatments, as assessed through luciferase emission (two-way ANOVA; P < 0.0001). Animals treated sequentially with GSK126 → BTZ had a significantly higher inhibitory response than animals that received single treatments and controls.

Next, we explored the impact of inflammatory signaling on cell proliferation and BTZ sensitivity in human cells to determine whether it would reveal similar vulnerability to this sequential treatment in vitro. KRAS mutant A549 and H1944 lung cancer cells were exposed to GSK126 or vehicle in vitro. We reseeded them into a 384-well format using either standard RPMI or IFN media. The inflammatory medium moderately impaired the proliferation of both A549 and H1944 cells under GSK126 treatment and imparted sensitivity to BTZ, regardless of Ezh2 inhibition (Fig. 8 B).

For an unbiased detection of the vulnerabilities of the KRAS mutant A549 under inflammatory conditions, we next performed a screen using 293 compounds from two independent libraries. These included kinase inhibitors and a National Cancer Institute (NCI)–approved oncology drug set. After 5 d, a CellTiter-Blue assay was used to determine cell viability. We screened two dosages (30 nM and 300 nM), and after verifying that most cytotoxic hits were GSK126 independent under our experimental conditions, we focused on the 300 nM dosage, which had a higher discovery potential. Remarkably, in both screens, the proteasome inhibitors Carfilzomib and BTZ were among the top inhibitory compounds (Fig. 8 C), a validation of our hypothesis-driven choice for sequential treatments. Surprisingly, five of the top ten compounds in the 300 nM screen targeted RNA synthesis, with BMS-387032 and flavopiridol (Alvocidib; the NCI-approved flavopiridol) specifically targeting CDK(9)-driven transcriptional initiation and elongation.

The results of our screen suggested that in the presence of inflammatory signaling, A549 cells might selectively rely on RNA synthesis. If that were the case, then RNA synthesis would represent a general mechanism that linked the inhibition of Ezh2 to the transcriptional amplification of inflammation (Fig. 8 D). To test this hypothesis, we treated two independent Kras-driven cell lines with GSK126 or vehicle for 5 d and then subjected them to low-confluence reseeding, which leads to NF-κB activation in the absence of external signaling (Fig. 4 E). To prevent toxicity, we limited the incubation period with BMS-387032, flavopiridol or vehicle to 4 h. Quantitative RT-PCR (RT-qPCR) showed that inhibiting RNA synthesis with both BMS-387032 and flavopiridol abrogated the GSK126-dependent induction of inflammatory mediators such as IL-6, Ccl2, and Il1ra in A549 (Fig. 8 D, lower left). Similar results were obtained in H1944 cells (Fig. 8 D, lower right). Levels of Irak1 were depressed by CDK inhibition even if not induced by GSK126, and the maintenance of a steady level of transcription of Tgfb1 supported RNA synthesis as a mechanism that was required specifically for the induction of inflammatory genes (Fig. 8 D).

In summary, our data suggest that Kras-driven NSCLC cells in the presence of Ezh2 inhibition and the transcription of inflammatory genes selectively rely on proteostasis and RNA synthesis to maintain cell fitness.

## Discussion

The idea that inhibiting PRC2 might be useful in fighting cancer has been around for over a decade (Varambally et al., 2002; Bracken et al., 2003; Kleer et al., 2003; Sparmann and van Lohuizen, 2006; Margueron and Reinberg, 2011). Correlations were found between Ezh2 expression and poor survival in several tumors, but specific inhibitors that target PRC2 have only shown success in a limited set of genotype-specific tumors that exhibit an addiction to polycomb activity (Sneeringer et al., 2010; McCabe et al., 2012; Mohammad et al., 2017). On the other hand, the recognition that PRC2 proteins have potent tumor-suppressive functions urges caution (Koppens and van Lohuizen, 2016); targeting PRC2 requires a detailed understanding of adaptive responses in vivo, and preclinical tumor models are appropriate systems in which this can be pursued. We have recently shown that the loss of PRC2 through Eed deletion in Kras-driven adenocarcinomas induces sterile inflammation. This leads to the non–cell-autonomous recruitment of tumor-associated macrophages, polarization, and EMT-mediated mucinous differentiation. This is an undesirable effect that occurred (depending on the p53 status of the tumors) when PRC2 is genetically inhibited in normal cells (Gargiulo et al., 2016; Serresi et al., 2016). Here, we show that using GSK126 to enzymatically inhibit PRC2 in tumor cells in vivo promotes inflammation and microenvironmental changes in orthotopic transplantation lung cancer models. Unlike the complete PRC2 loss during tumor initiation, pharmacological inhibition seems to elicit a cell-cycle restriction also in the most aggressive Kras*;Trp53* background, and, importantly, it does not lead to the development of molecular features such as EMT and mucinous differentiation, indicators of tumor progression, that have been observed in the autochthonous models. Whether this depends on the tumorigenesis phase (progression vs. initiation), mode of inhibition (enzymatic interference vs. complete deletion), the duration and the extent of the functional inhibition of PRC2, and/or an involvement by components of the tumor microenvironment is an open question.

In this study, we characterized the response to Ezh2 inhibition, which entails the amplification of a transcriptional program involving NF-κB. In turn, this inflammatory response rewires Kras-driven tumors at the molecular level, leading both to the activation of a pro-oncogenic and prometastatic program and to an increase in vulnerability to treatments that combine BTZ and NM. We propose that treating Kras-driven NSCLC by applying GSK126 in a neoadjuvant-like manner enhances the tumor’s susceptibility to subsequent treatments that can be defined experimentally. Here, we perturbed proteostasis and inflammation using small molecules. The combination of BTZ/NM after neoadjuvant GSK126 treatment in carcinomas that homogeneously express Ezh2 has pro-apoptotic effects alongside modifications of the tumor microenvironment (Fig. 7 K). Precisely assessing the complex interactions between the drugs in the sequential dosing experiments and their impact on the overall survival will benefit of additional tumor models such as autochthonous, syngeneic, and/or xenografts models.

Phase 3 clinical trials are currently underway using BTZ as a therapy to treat NSCLC (i.e., NCT01833143), and second-generation proteasome inhibitors are being applied in phase 4 trials (e.g., NCT03416374). A hypothesis emerging from the current study suggests that a combination of proteasome inhibitors with other small molecules targeting other associated dependencies, such as inflammation and RNA synthesis (e.g., Cox2 and CDK9 inhibitors, respectively) should be pursued at preclinical level and compared with the BTZ and NM combination.

Since the inhibition of Ezh2 has recently been shown to amplify TNF-α–induced NF-κB signaling and inflammation in normal intestinal epithelium (Liu et al., 2017), it is plausible that our findings in lung cancer can be extended to other Kras-driven epithelial malignancies, such as pancreatic ductal carcinoma. Future studies should be aimed at the systematical characterization of the source of upstream signaling of the pathways that we identified as potentially cooperating with Ezh2 inhibition to drive the transcriptional amplification and GSK126-dependent microenvironmental changes (e.g., TNF-α and JAK/STAT). The difference in inflammatory signaling as cell intrinsic or non–cell autonomous will potentially determine whether immunotherapy can be a major target. Evidence in support of a tight interplay between cancer epigenetics and immunology is rapidly accumulating (Miao et al., 2018; Pan et al., 2018), and immunotherapy effectiveness as monotherapy is so far restricted to a small set of lung cancer patients (Rizvi et al., 2015). In our experimental setting, GSK126 induces CD47 and the expression of its cognate receptor SIRPA, which has been identified as oncogene in the in vivo RNAi screen. Interestingly, CD47 immunomediated depletion proved to be effective in preclinical models for NSCLC and evidence indicates a mechanism involving enhanced phagocytosis (Kojima et al., 2016; Weiskopf et al., 2016). The identification of a mast cells activation signature in a transplantation-based mouse model may reflect the outcome of a BALB/c-specific microenvironment. Moreover, Ezh2 inhibition also affects macrophages response to inflammatory signaling (Zhang et al., 2018). Hence, testing the hypothesis involving neoadjuvant-like Ezh2 inhibition followed by immunotherapy, such as therapeutic targeting of the CD47/SIRPA axis, should be extended to relevant autochthonous models with an intact innate immune system in which PRC2 loss causes inflammation (Serresi et al., 2016; Gargiulo, 2018). The major outcome of this study is to demonstrate that orthotopic grafting of *Kras^G12D^*-driven NSCLC promotes homogeneous Ezh2 expression, whose inhibition in vivo unleashes an inflammatory response. During tumor progression, Ezh2 inhibition amplified vulnerabilities associated with acute inflammatory transcription as revealed by sensitivity of treated tumor cells to inhibition of the cellular machineries involved in proteostasis and RNA synthesis and, indirectly, of the NF-κB pathway. More in general, we explored a paradigm of targeting the development of adoptive resistance in cases in which preclinical testing supports the use of a specific sequential treatment. One example of this approach has been reported in the case of secondary mutations in the MEK–ERK pathway in vemurafenib-resistant BRAF-mutant melanomas (Wang et al., 2018). The successful generalization of these approaches and systematic clinical application will require the identification of predictive biomarkers.

## Materials and methods

### Mouse strains

All experiments involving animals complied with local and international regulations and ethical guidelines and were authorized by our local experimental animal committee at the Netherlands Cancer Institute.

K-rasLSL-*^G12D/+^* (Jackson et al., 2001) and p53F2-10/F2-10 (Jonkers et al., 2001) mice have been previously described (Xie et al., 2014).

### Intratracheal and intravenous transplantation

7- to 9-wk-old BALB/c nude mice were intratracheally intubated with 10^6^ cells per 20 μl PBS. For tail vein injections, cells were resuspended in 50 μl PBS + 50 μl saline solution. A detailed description of the procedure has been published before (Gargiulo et al., 2014).

### Cell lines

A549, H1944, H2030, and H2122 human cell lines (provided by the R. Bernards laboratory, the Netherlands Cancer Institute, Amsterdam, the Netherlands) were cultured in RPMI medium with 10% FBS and 5% penicillin and streptomycin at 37°C in a 5% CO_2_–95% air incubator. Primary mouse NSCLC Kmix and KP cell lines were isolated from a *Kras^G12D/+^*; and *Kras^G12D/+^;Trp53*^−/−^ genetic background, respectively. Cells were isolated and propagated in F12 medium supplemented with 10% FBS, 5% penicillin and streptomycin, hydrocortisone, recombinant EGF, insulin-transferrin selenium. Raw267.4 cells (ATCC) were cultured in DMEM medium with 10% FBS and 5% penicillin and streptomycin at 37°C in a 5% CO_2_–95% air incubator, except when co-cultured with KP cells, in which case they were propagated in KP medium.

### Expression and knockdown constructs

For Ezh2 knockdown experiments, we used dox-inducible FH1-tUTG-RNAi vectors (Herold et al., 2008), as described previously (Michalak et al., 2013), containing the following targeting sequences: Ezh2-tetKDa, 5′-GCAAAGCTTGCATTCATTTCA3′; Ezh2-tetKDb, 5′-GCAACACCCAACACATATAG-3′.

For Eed knockdown experiments, we used dox-inducible pLKO.1-Tet-ON containing the following targeting sequences: Eed#A5, 5′-GAAGCAACAGAGTAACCTTAT-3′; Eed#B4, 5′-CGGCTATTCGACAAACCAGTT-3′.

We also performed ablation experiments by using siRNA smart pools against mouse EZH2 (ON-TARGETplus mouse EZH2 siRNA L-040882; ON-TARGETplus non targeting pool D-001810-10-20). VAV1, CXCL1, and CXCR4 overexpression constructs were from the CCSB-Broad Lentiviral Expression Library in the pLX304-Blast-V5 vector. pLKO.1-CMV-L1CAM-puro was provided by the M. Tsao laboratory (Ontario Cancer Institute, Toronto, Canada) and the luciferase vector by the D. Peeper laboratory (Netherlands Cancer Institute, Amsterdam, Netherlands).

### In vitro and in vivo drug treatment

For in vivo experiments, BALB/c mice (Charles River) were superior to NMRI nude owing to a better response to GSK126. For an MTD, BALB/c nude mice received a single treatment via i.p. NM (Sigma) and BTZ (LC Labs) in PBS (0.5% DMSO) at 5–1 mg/ kg body weight, respectively. To reduce the impact of multiple cycles of treatment, BTZ was dosed at 0.8 mg/kg the first day and 0.2 mg/kg body weight on the second day, in which 5 mg/kg NM was also given.

IVIS Lumina imaging was performed as described previously (Gargiulo et al., 2014).

In vitro, NSCLC cells (10^5^ per well) were seeded into 6-well tissue culture plates in the appropriate cell culture media 24 h before treatment. To induce EZH2 or EED knockdown, cells were treated with dox continuously for 5 d. To perform clonogenic assays, cells treated with dox for 5 d were replated at 250 or 500 cells per well and kept in dox for the duration of the entire experiment. To achieve H3K27 trimethylation inhibition, cells were exposed to 0.1% DMSO (vehicle) or 5 µM GSK126 for at least 6 d. GSK343 was used at 100 nM concentration for 5 d. Western blot analysis was performed using standard methods. Whole-cell extracts were prepared in RIPA buffer (50 mM Tris, pH 8.0, 50 mM NaCl, 1.0% NP-40, 0.5% sodium deoxycholate, and 0.1% SDS) containing protease inhibitor cocktail (Complete; Roche) and phosphate inhibitors (10 mM Na fluoride final concentration, 1 mM sodium orthovanadate final concentration, and 1 mM NaPPi final concentration). Equal amounts of protein, as determined by a Micro BCA Protein Assay Kit (Pierce), were resolved on NuPage-Novex 4–12% Bis-Tris gels (Invitrogen) and transferred onto nitrocellulose membranes (0.2 m; Whatman). Membranes were blocked in PBS 0.1% Tween-20 (PBST) 5% BSA for 1 h, incubated with primary antibodies in PBST 1% BSA overnight at 4°C, and incubated with secondary antibodies coupled to HRP for 45 min in PBST 1% BSA. Bands were visualized using enhanced chemoluminescence Western blotting detection reagent (GE Healthcare). Primary antibodies used against the following antigens were anti-NFKBSer536 (3033; Cell Signaling), anti–total NFKB p65 (C22B4 4764; Cell Signaling), anti-vinculin (clone h-VIN1 V9131; Sigma), anti-H3K27me3 (9733; Cell Signaling), anti-EZH2 (612666; BD), anti–phospho-AKT Ser473 (D9E 4060; Cell Signaling), total AKT (9272; Cell Signaling), pospho-Stat3 Y705 (D3A7 9145L; Cell Signaling), anti-PARP (9542; Cell Signaling), anti-BMI1 (F6 clone; made in-house), phospho-Erk (9101; Cell Signaling), and total Erk (4695; Cell Signaling).

The cell cycle was measured by a BrdU pulse (0.2 mg/ml, B9285; Sigma-Aldrich) given 60 min before ethanol fixation. FACS analysis was performed after incubation in the dark with anti–BrdU-FITC (Becton Dickinson) and propidium iodide (5 μg/ml; Sigma), in the presence of RNaseA (5 mg/ml).

Luminescence and fluorescence were acquired and quantified using a TECAN SPARK 20M plate reader with standard settings for luminescence, and monochromator excitation/emission, respectively. In all cases, P values were then calculated using Prism.

### RT–qPCR and RNA-seq

RNA was isolated using Trizol and cDNA was generated using SuperScript II or VILO according to the manufacturer’s instructions (Invitrogen). Primer details are available upon request. For RNA-seq, the library was prepared using TruSeq RNA sample prep kit according to the manufacturer’s protocol (Illumina). RNA-seq data were deposited in the Gene Expression Omnibus (GEO) under accession no. GSE61125.

### IHC

For histological analysis, lungs were inflated with formalin and fixed for 24 h in formalin. Fixed tissues were subsequently dehydrated and embedded in paraffin, and 2- to 10-µm sections were prepared and stained with H&E.

We performed IHC for anti-EZH2 (612666; BD), anti-H3K273me (9733; Cell Signaling), anti-GFP (AB6566; AbCam), anti–phospho-p44-p42 (rabbit polyclonal 4370; Cell Signaling), anti-CXCR4 (NB100-74396; Novus Biologicals), anti-VAV1 (NB100-92004; Novus Biologicals), anti-CEPB β (C-19:sc-150; Santa Cruz), and anti–cleaved caspase (Asp175, 9661; Cell Signaling). Chromogen system (K3468; DAKO) and Powervision Poly-HRP (Leica Microsystems) were used for IHC detection. Immunofluorescence used species-specific Alexa Fluor Plus 647 (Thermo Fisher).

### Image quantification

Tumor burden and H3K27me3/DAPI stainings were quantified using ImageJ software. The tumor area size was calculated for each H&E section by inverting channels until only tumor and no normal lung tissue was colored. Automatic quantification was performed. A similar approach has been used to calculate colony size. For immunofluorescence quantification, all images were subjected to the same procedure. Briefly, we imported images in ImageJ, converted to 32-bit; the signal was inverted and thresholding used “default” and “AUTO” profiles, and the signal was identified by “Watershed” function and region of interest parameters as follows: size = 0.001 to infinity; circularity = 0.1 to infinity. Analyses were performed on the integrated density data (IntDen).

In all cases, P values were then calculated using Prism.

### In vivo RNAi screen

The RNAi screen was designed and performed as previously described in detail (Gargiulo et al., 2013, 2014). Briefly, a custom-designed library of pLKO.1-encoding shRNAs was assembled from the Broad Institute public library. Lentiviral supernatants infections were performed as described in the references above, and NSCLC cells were infected separately by one round of overnight exposure to the viral pool. Multiplicity of infection was experimentally designated as <1 due to roughly 50% cell death upon 1 µg/ml puromycin selection for cells expressing integrated shRNA. 0.8–2 × 10^6^ cells were intravenously injected in the tail vein, and successful grafting was assessed via noninvasive bioluminescence. On the onset of shortness of breathing, animals were sacrificed and lung tumors dissected to obtain a single cell suspension. BD FACSAria III (BD Bioscience) was used to sort out GFP-positive tumor cells. The genomic DNA was isolated using DNeasy tissue and blood kit (Qiagen). 2 μg of DNA was used to perform individual shRNA amplification and purification used AMPure beads (Ambion). Purified PCR2 products were ready for Illumina sequencing and sequenced using HiSeq2000 to identify the shRNA. Reads were generated in individual tumors and counts were log2 transformed, followed by normalization to the average of four input samples for individual reads. Data were plotted as fold change versus input on the y axis and as normalized abundance (the ratio between individual shRNA counts and total read count per tumor sample). Outlier identification was performed in Prism using standard xy plots. A strict fold-change threshold used was to balance the less conservative parameters used in Outlier identification (Gargiulo et al., 2013, 2014).

For both EdgeR and DESeq2 analyses of statistically significant hits, we used Seqmonk. Significance by read count quantitation using all reads was determined as comparison versus input, with a P < 0.05 after Benjamini–Hochberg correction and ±1(log2) fold change. A custom genome was created with individual probes named after individual TRC shRNAs. Seqmonk raw and normalized counts can be found in Table S1. PCA in Fig. 6 B was performed on all quantitated probes after correcting for total count to largest store log transformed.

### Drug screen with compound libraries

The screen was performed with the Selleck Kinase Inhibitor Library as well as the NCI Oncology set. To aim at a final concentrations of 30 and 300 nM, we first created DMSO master stock plates of 30 and 300 µM, respectively. From these, 100× dilution in IFN medium (see below) was obtained first, followed by a further 10× to the assay plates (final 1,000×, 0.1% DMSO). The concentration of 250 cells per well was experimentally determined beforehand to take into account proliferation rates over 5 d and response to cytotoxicity. A549 cells were seeded in 384-well Corning black clear-bottom plates using the Multidrop Combi (Thermo Scientific) after being resuspended in RHB-A (Takara), supplemented with the following cytokines: Heparin-binding EGF (HB-EGF; 0.4 µM), NGR1 (3.7 µM), TGF-α (3.8 µM), IL-6 (1.2 µM), TNF-α (0.9 µM), IFN-γ (0.9 µM), and LPS (4 µM). After 24 h, the combined NCI-approved oncology drug and Selleck libraries set were added using the MICROLAB STAR liquid handling workstation (Hamilton). The libraries were stored and handled as recommended by the manufacturer. From the master plate, 10 mM stocks were diluted to the final concentrations in daughter plates containing RHB-A medium. From daughter plates, compounds were transferred into 384-well assay plates, in triplicate, with final concentrations of 300 nM and 30 nM. Compounds were refreshed 48 h later. After 5 d, medium was replaced and cells were incubated for 24 h with fresh RHB-A supplemented with HB-EGF (0.4 µM), and a CellTiter-Blue assay (Promega) was performed as recommended by the manufacturer. Fluorescence intensity was measured using the Envision Multilabel Plate Reader with 544-nm excitation and 590-nm emission filters. The cellHTS2 application was used to normalize the data. Data were normalized per plate using the normalized percentage inhibition method; the mean of the positive control (BTZ) was set to 0, and the mean of the negative control was set to 1. Per compound and concentration, the mean was determined over the three replicates. The screenings were retained for further analyses after verification that the z′factor > 0. Ranking for relative viability was based on cumulative frequency distribution and the cutoff for Top10 drugs was arbitrary. The full screen results can be found in Table S2. Mubritinib (TAK-165) was the most significantly GSK126-regulated compound based on a Student’s *t* test. Mubritinib, TAK-733, flavopiridol, and BMS-387032 were all independently purchased (Selleck) and validated.

### Bioinformatic analyses

RNA-seq reads were mapped to the transcriptome using Tophat. Gene expression values were determined using HTseq-count. In each sample, unique reads were aligned to the reference genome, and reads mapping to multiple locations were discarded. Absolute expression values were normalized to 10 million reads followed by log2 transformation. Lowly expressed genes were excluded before DRGs were called out. For instance, down-regulated genes were called out when showing at least a count-per-million normalized value of >7 and a fold change >1.1. ChIP-seq data were obtained from the GEO (accession no. GSE61125). Using the seqMINER 1.3.2 platform, data were subsequently clustered with respect to a list of unique RefSeq genes. Heat maps and the average profiles of read density for each cluster were generated. We used Kmeans linear as the method of clustering, with the following parameters: left and right extension = 5 kb, internal bins (with respect to the gene body) = 120, and number of cluster = 7.

Expression array clustering was performed using the library “gplots” in R. “heatmap.2” function has been used to generate the heat maps and samples has been clusterized by Spearman correlation.

The clustering analysis in Fig. 3 J was performed using MeV4.8 and Pearson correlation. The input DRGs were identified using Seqmonk. Briefly, we imported all the .bam files and annotated them to the mouse genome (GRCm38). The most stringent filter in DESeq2/EdgeR was used at P < 0.02 after Benjamini–Hochberg correction. Quantitation was performed using RNA-seq pipeline quantitation on merged transcripts counting reads over exons correcting for DNA contamination, assuming a non–strand-specific library. The same procedure in Seqmonk was used to generate the highlighted gene sets in the MA plot in Fig. 4 C. ChIP-seq analysis was performed with Seqmonk, and CpG Island annotation was extended ±5 kb on either side. P values for gene set enrichment analysis were generated with the standard gene set filter in Seqmonk at P < 0.05 and using correction for multiple comparisons. Inputs for the analysis (I58 and I59) were the same in all comparisons and were imported from the GEO (accession no. GSE61125). The nearest annotated genes to CGIs were used to generate the overlap between K27me3+ CGI and DRGs in Fig. 4 A using Venny.

In Fig. 4 (A and D), we determined HuE regulated genes using EdgeR with a significance below 0.02 after Benjamini–Hochberg correction on merged transcripts counting reads over exons as raw counts and assuming a non–strand-specific library. This returned 8,554 gene, which were reduced to 6,241 by applying a filter for relative expression. Finally, we applied a fold-change of >1.5 (log2) over vehicle to identify the final set of 832 up-regulated genes. The overlapping up-regulated genes with H3K27me3 targets from orthotopic NSCL were 176 out of 2,492 (or 176/832). We visualized the same set of genes in all the four plots in Fig. 4 D. Using the same criteria in Fig. 4 A, 225 genes were up-regulated in OS50 (100/225 were K27me3) and only three in the cells treated in presence of LPS + GSK126 KP.

CIBERSORT analysis was performed with standard settings, and P < 0.001. The number of iterations (either 100 or 1,000) did not alter the result of the analysis.

PROGENy analysis used the pipeline described by the authors (Schubert et al., 2018). Mouse genes were used as input after filtering from the original precomputed matrix. KisSplice (Lopez-Maestre et al., 2016) allele frequency (Fig. 5, C and D) was generated by following the pipeline for nonreference genome described at http://kissplice.prabi.fr/TWAS/. The reference genome GRCm38. SNP information for the donor/vehicle mice strain was obtained from the Jax database (http://www.informatics.jax.org/). BALB.cByJ and C57BL.6J SNP data were downloaded from http://www.informatics.jax.org/downloads/.

Correlation, graphical representation and posteriors analysis were done using R. The frequency distributions in Fig. 5 E were calculated from the allele frequency output of the KisSplice procedure. Likewise, the representation in Fig. 5 F was based on the same procedure, except that input genes were restricted to a significant IPA category based on the IPA core analysis of all BALB/c-specific SNPs.

### Dose response of NSCLC cells to BTZ under inflammatory conditions

A549 and H1944 cells were seeded at low confluency in 384-well black-walled clear-bottom microplates (Greiner) in either RPMI1640 + 10% FBS + 1% Penstrep or IFN medium (RPMI1640 plus 0.4 µM HB-EGF, 3.7 µM NGR1, 3.8 µM TGF-α, 1.2 µM IL-6, 0.9 µM TNF-α, 0.9 µM IFN-γ, and 4.0 µM LPS) with and without the addition of 5 µM GSK126. After 24 h, BTZ was added in a dosage range of 0.01 to 300 nM using the TECAN d300e compound printer, including four replicates per dosage condition with randomized well distribution and DMSO normalization. GSK126 treatment was renewed 24 h after the application of BTZ using the compound printer. Viability readout was performed 72 h after drug application using the AlamarBlue assay (Sigma) according to the manufacturer’s protocol. Fluorescence intensity values were acquired using a TECAN SPARK 20M plate reader with standard fluorescence top reading settings and monochromator excitation 565 nm (10 nm)/emission 592 nm (10 nm). GraphPad PRISM was used for subsequent data analysis. Concentration values in [M] were log transformed, and means of fluorescence intensity values of untreated wells were used to normalize raw data and plot data points as percentage of viability. A nonlinear regression fit (log(inhibitor) vs. normalized response–variable slope) was used to determine IC_50_ values. P values were calculated using one-way ANOVA with post-hoc testing of significance.

### Database information

The RNA-seq data generated in this study are available at the GEO under accession no. GSE61125. The ChIP-seq and RNA-seq data generated in a published study were previously deposited to the GEO under accession no. GSE61190. Tumor samples with mRNA data (RNA Seq V2) for human lung squamous cell carcinoma (TCGA, provisional, 501 samples) were analyzed at http://www.cbioportal.org.

### Online supplemental material

Fig. S1 shows that Eed depletion impairs *Kras^G12D/+^;Trp53*^−/−^ cell colony formation and that this effect is rescued by TNF-α and LPS signaling. Table S1 contains the parallel in vitro/in vivo RNAi screen read counts. Table S2 contains normalized compound screening data.

## Supplementary Material

Supplemental Materials (PDF)

Table S1 (XLSX)

Table S2 (CSV)
