# Supplementary material for: Ezh2 inhibition in Kras-driven lung cancer amplifies inflammation and associated vulnerabilities
Source: J Exp Med. 2018 Dec 3;215(12):3115–35. doi: 10.1084/jem.20180801 (PMC6279402; doi:10.1084/jem.20180801)
Supplement: Supplemental Materials (PDF) [file JEM_20180801_sm.pdf]

Supplemental material

Serresi et al., https://doi.org/10.1084/jem.20180801

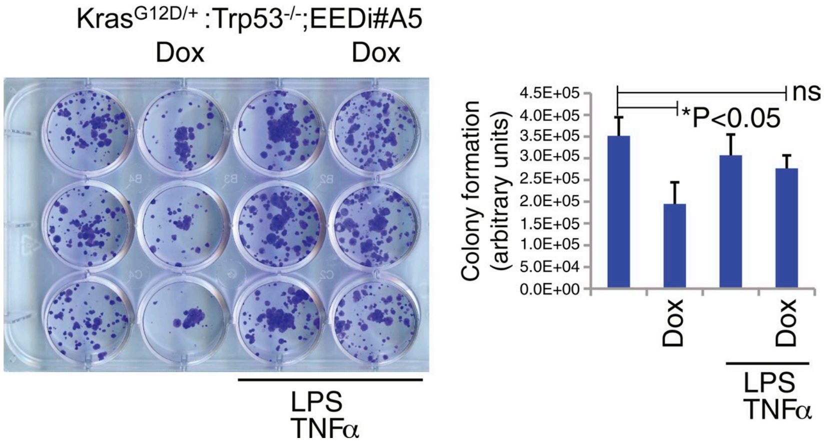

Figure S1. **Acute inflammatory signaling rescues Eed depletion.** Left: NSCLC cells were cultured as indicated, and colony formation of 250 cells per well was assessed. Right: ImageJ quantification. Error bars indicate SD. P value was calculated by ANOVA and Holm–Sidak's post-test.

Provided online are two Excel files. Table S1 contains parallel in vivo/in vitro RNAi screen data, and Table S2 contains compound screen data.
